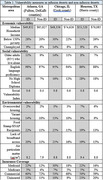# Social and economic vulnerability in metropolitan areas with limited access to infusion centers in the US

**DOI:** 10.1002/alz70858_103347

**Published:** 2025-12-26

**Authors:** Shardae Showell, Cai Gillis, Lauren Powell

**Affiliations:** ^1^ Biogen, Cambridge, MA, USA

## Abstract

**Background:**

After decades of waiting, two amyloid directed immunotherapies for AD provide hope for patients. However, social vulnerability and a lack of easily accessible infusion centers may unduly limit those living with AD from receiving treatment. Despite the abundance of healthcare services in U.S. metropolitan areas, systemic, economic, and social challenges leave many marginalized communities underserved. Here we explored how social factors relate to access to infusion centers in metropolitan areas.

**Method:**

Metropolitan areas with racial (>45% non‐white) and/or ethnic (>20% Hispanic/Latino) diversity across the US were selected (Atlanta, GA; Chicago, IL; and Houston, TX) for examination of infusion deserts (IDs) using National Infusion Center Association Locator Data to identify areas with >5 miles radius to the nearest infusion center. Social, economic, and environmental characteristics between IDs and non‐IDs were compared using CDC PLACES data and the Agency for Healthcare Research and Quality Social Determinants of Health Database.

**Result:**

IDs showed greater social, economic, and environmental vulnerability compared to non‐IDs. Median household income ranged from 24%‐42% lower in IDs with a higher proportion of households living below the 150% Federal Poverty Level than in non‐IDs. In Atlanta and Chicago, IDs had a 3‐4% higher proportion of vacant housing than non‐IDs, but in Houston proportions were similar between IDs and non‐IDs. Older adults in IDs were more likely to live alone than older adults in non‐IDs (Table 1).

IDs had a higher proportion of those who were unemployed, food stamp/SNAP recipients, and lacked a high school diploma; and were less likely to have broadband access compared to non‐IDs. Insurance coverage differed, with non‐IDs having a larger proportion of residents on commercial insurance compared to IDs; IDs showed larger proportions of residents uninsured or on Medicaid compared to non‐IDs.

**Conclusion:**

IDs showed greater economic and social vulnerability among residents compared to non‐IDs. Older adults were more likely to live alone in IDs, potentially compounding difficulty in accessing disease modifying therapies for early AD. These challenges prevent many individuals from accessing breakthrough AD therapies, which are crucial for improving health outcomes in disproportionately affected communities.